# Divergent patterns of telomere shortening in tropical compared to temperate stonechats

**DOI:** 10.1002/ece3.4769

**Published:** 2018-12-26

**Authors:** Beate Apfelbeck, Mark F. Haussmann, Winnie Boner, Heiner Flinks, Kate Griffiths, Juan Carlos Illera, Kim G. Mortega, Zachary Sisson, Patrick Smiddy, Barbara Helm

**Affiliations:** ^1^ Institute of Biodiversity, Animal Health and Comparative Medicine University of Glasgow Glasgow UK; ^2^ Terrestrial Ecology Research Group, Department of Ecology and Ecosystem Management, School of Life Sciences Weihenstephan Technische Universität München Freising Germany; ^3^ Department of Biology Bucknell University Lewisburg Pennsylvania; ^4^ Borken Germany; ^5^ Research Unit of Biodiversity (UO‐CSIC‐PA), Oviedo University Mieres Spain; ^6^ Department of Migration and Immunoecology, Max‐Planck‐Institut für Ornithologie Radolfzell Germany; ^7^ Museum für Naturkunde—Leibniz‐Institut für Evolutions‐und Biodiversitätsforschung Berlin Germany; ^8^ School of Biological, Earth and Environmental Sciences University College Cork Cork Ireland; ^9^ Groningen Institute for Evolutionary Life Sciences (GELIFES) University of Groningen Groningen The Netherlands

**Keywords:** extended parental care, life history, pace of life, selective disappearance, telomeres, tropical and temperate environments

## Abstract

Telomeres have emerged as important biomarkers of health and senescence as they predict chances of survival in various species. Tropical birds live in more benign environments with lower extrinsic mortality and higher juvenile and adult survival than temperate birds. Therefore, telomere biology may play a more important role in tropical compared to temperate birds. We measured mean telomere length of male stonechats (*Saxicola *spp.) at four age classes from tropical African and temperate European breeding regions. Tropical and temperate stonechats had similarly long telomeres as nestlings. However, while in tropical stonechats pre‐breeding first‐years had longer telomeres than nestlings, in temperate stonechats pre‐breeding first‐years had shorter telomeres than nestlings. During their first breeding season, telomere length was again similar between tropical and temperate stonechats. These patterns may indicate differential survival of high‐quality juveniles in tropical environments. Alternatively, more favorable environmental conditions, that is, extended parental care, may enable tropical juveniles to minimize telomere shortening. As suggested by previous studies, our results imply that variation in life history and life span may be reflected in different patterns of telomere shortening rather than telomere length. Our data provide first evidence that distinct selective pressures in tropical and temperate environments may be reflected in diverging patterns of telomere loss in birds.

## 1 INTRODUCTION

Variation in life histories is thought to result from differential allocation of limited resources to competing life history traits. Such trade‐offs and the resulting optimal resource allocation may vary with environmental conditions (Stearns, 1992). For example, tropical environments have favored a slow pace of life, that is, reduced fecundity but increased life span, in many vertebrates (Ricklefs & Wikelski, 2002). This is especially well studied in birds where tropical species produce fewer, but higher quality offspring (Jetz, Sekercioglu, & Böhning‐Gaese, 2008; Martin, 2015), have lower basal metabolic rates (Tieleman et al., 2009; Wiersma, Muñoz‐Garcia, Walker, & Williams, 2007) and live longer (Møller, 2007; Peach, Hanmer, & Oatley, 2001) than temperate species. Therefore, a comparison between tropical and temperate species may reveal physiological constraints that may limit the evolution of alternative combinations of life history traits (Ricklefs & Wikelski, 2002).

An important candidate mechanism with respect to physiological constraints of growth, reproduction and survival are telomeres (Haussmann & Marchetto, 2010). Telomeres are noncoding DNA—protein caps at the end of eukaryotic chromosomes that protect genomic integrity, but shorten during cell division and potentially when exposed to oxidative stress (Boonekamp, Bauch, Mulder, & Verhulst, 2017; Reichert & Stier, 2017; Zglinicki, 2002). Critically, short telomeres eventually lead to cell senescence or death (Blackburn, 2000, 2005), and the accumulation of cells with short telomeres may be one of the factors that causes aging and senescence in vertebrates (López‐Otín, Blasco, Partridge, Serrano, & Kroemer, 2013).

Both longitudinal and cross‐sectional studies in birds show that, in general, older individuals have shorter telomeres than younger ones with the greatest loss in telomeres occurring early in life (Heidinger et al., 2012; Pauliny, Larsson, & Blomqvist, 2012; Salomons et al., 2009; Spurgin et al., 2018; Tricola et al., 2018). Furthermore, an increasing number of studies in birds show that individuals with longer telomeres or little telomere attrition have better survival prospects than individuals with short telomeres or high levels of telomere attrition (reviewed in Wilbourn et al., 2018). This has been especially well studied in zebra finches (*Taeniopygia guttata*), for which it has been shown that long telomeres in early life are associated with increased survival and a long life span (Heidinger et al., 2012). In addition, studies in a variety of species show that telomere dynamics are sensitive to environmental influences such as variations in food availability (Spurgin et al., 2018), parasitic diseases (Asghar et al., 2015), and exposure to stress (Hau et al., 2015). In particular, conditions experienced during development can influence telomere dynamics. For example, exposure to poor or stressful environments can lead to accelerated telomere loss in young birds (Costanzo et al., 2017; Haussmann, Longenecker, Marchetto, Juliano, & Bowden, 2012; Herborn et al., 2014; Nettle et al., 2017; Salmon, Nilsson, Nord, Bensch, & Isaksson, 2016; Soler et al., 2017; Young et al., 2017), which can be predictive of decreased survival as nestlings or fledglings (Boonekamp, Mulder, Salomons, Dijkstra, & Verhulst, 2014; Salmon, Nilsson, Watson, Bensch, & Isaksson, 2017; Watson, Bolton, & Monaghan, 2015). Thus, telomere length and the rate of telomere loss are considered biomarkers of individual health and quality (Young, 2018).

Fewer studies have compared telomere length between taxa that vary in their life histories and life span. In mammals, a comparative study found that short‐lived, small species have longer telomeres and higher telomerase expression than long‐lived, large species (Gomes et al., 2011). In a study on rodents, no relationship between maximum lifespan and telomere length was detected (Seluanov et al., 2007). In birds, absolute telomere length does not seem to relate to variation in lifespan between species; however, longer‐lived avian species seem to have lower rates of telomere shortening than shorter‐lived species (Dantzer & Fletcher, 2015; Haussmann et al., 2003; Sudyka, Arct, Drobniak, Gustafsson, & Cichoan, 2016; Tricola et al., 2018). This relationship between rate of telomere loss and maximum lifespan in birds may be caused by variation between species in how well telomeres are maintained throughout their lifespan. In addition, it may reflect selective disappearance of low‐quality individuals with short telomeres. In longer‐lived species, that experience lower levels of extrinsic mortality, individual condition, and thus telomere dynamics, may play a greater role as determinants of mortality (Kirkwood & Austad, 2000). Therefore, selective disappearance of individuals with short telomeres may be more apparent in long‐lived species (Tricola et al., 2018).

Tropical species live in less seasonal environments with lower levels of adult extrinsic mortality than temperate ones (Brown, 2014). Consequently, tropical songbirds have higher survival probabilities than temperate birds (Martin et al., 2017; Muñoz, Kéry, Martins, & Ferraz, 2018). Therefore, stronger selective disappearance of individuals with short telomeres is expected in tropical compared to temperate birds. However, mortality rates are age‐specific, and therefore, the strength of selective disappearance may vary with age. In birds, mortality is usually highest during the first year of life, especially directly after fledging (Cox, Thompson, Cox, & Faaborg, 2014; Naef‐Daenzer & Grüebler, 2016). As predicted by life history theory (McNamara, Barta, Wikelski, & Houston, 2008), juvenile survival is in general higher in tropical compared to temperate birds (Lloyd, Martin, & Roskaft, 2016; Remes & Matysiokova, 2016). Tropical parents take care of their fewer fledglings for considerably longer than temperate birds and may thereby be able to lower extrinsic mortality in juveniles (Styrsky, Brawn, & Robinson, 2005). We, therefore, hypothesize that differential survival of high‐quality fledglings should be more apparent in tropical compared to temperate birds. Assuming that telomeres are bioindicators of somatic state and individual quality we expect that in tropical birds, individuals with short telomeres disappear faster from a population than in temperate birds both during the critical first year of life and later as adults.

In addition, there is good evidence that tropical species invest more into self‐maintenance, but are less fecund than temperate species. For example, tropical species exhibit stronger sickness behavior after infection during the breeding season than temperate species (Owen‐Ashley, Hasselquist, Raberg, & Wingfield, 2008). Furthermore, in tropical species, reproductive workload is reduced as they lay smaller clutches, thereby caring for fewer young and expending less energy than temperate birds (Nilsson, 2002; Tieleman et al., 2006). Thus, they may reduce high levels of oxidative stress (Noguera, 2017) and potential telomere loss associated with breeding (Reichert et al., 2014). In addition, tropical songbirds seem to have lower post‐natal metabolic rates and slower, more sustained growth despite similar nestling times than temperate birds (Martin, 2015; Ton & Martin, 2016). These slower growth trajectories in combination with increased parental care per offspring may favor lower levels of telomere attrition during early life in tropical birds, which in turn may be important determinants of their longer life spans (Monaghan & Ozanne, 2018). Thus, longer‐lived tropical species, which invest more in growth and self‐maintenance than in fecundity, are expected to show longer telomeres as nestlings or a slower rate of telomere loss than short‐lived temperate species. To determine how life history variation has shaped variation in telomeres, comparisons between the same or closely related species in different environments are necessary.

**Figure 1:**
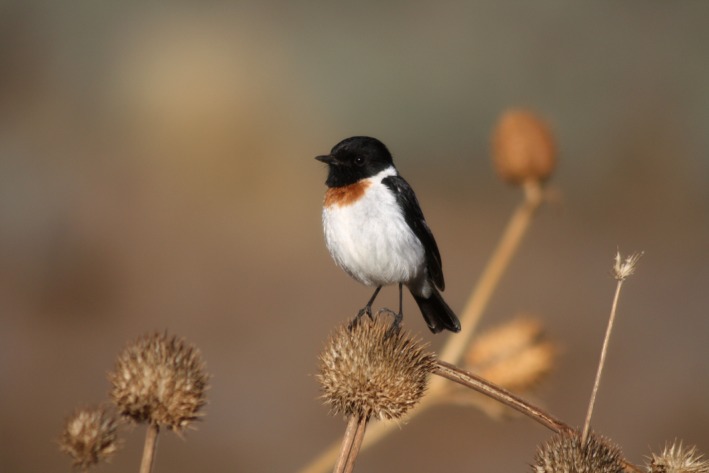
Adult male African stonechat

Here, we compare telomere length of two closely related sister taxa of stonechats, *Saxicola torquatus axillaris *(Figure 1) and *Saxicola rubicola *that breed in tropical and temperate environments, respectively (Doren et al., 2017; Urquhart, 2002). At all latitudes, stonechats are socially monogamous, open habitat, insectivorous passerines that aggressively defend breeding territories (Apfelbeck, Mortega, Flinks, Illera, & Helm, 2017). However, they vary in pace of life according to their environment (Ricklefs & Wikelski, 2002). Stonechats show a latitudinal cline in metabolic rate, with genetically inherited, higher metabolic rates in higher‐latitude populations (Klaassen, 1995; Tieleman et al., 2009; Versteegh, Schwabl, Jaquier, & Tieleman, 2012; Wikelski, Spinney, Schelsky, Scheuerlein, & Gwinner, 2003). Further, temperate stonechats have a genetically fixed larger clutch size than tropical ones (Gwinner, König, & Haley, 1995), and their higher fecundity correlates with elevated baseline corticosterone concentrations during the breeding season (Apfelbeck, Helm, et al., 2017). In agreement with lower adult extrinsic mortality in tropical environments, local survival of tropical stonechats appears to be much higher than that of temperate stonechats. In previous studies, the local apparent annual survival of stonechats in East Africa varied between 65% and 85% (Scheuerlein, 2000), while in a European population, it was only 29%–45% (Flinks, Helm, & Rothery, 2008; Flinks & Pfeifer, 1984).

We collected samples from Afrotropical (referred to as tropical) and temperate European (referred to as temperate) male stonechats from different individuals at four age classes: as nestlings, as pre‐breeding first‐year birds (within their first 6 months of life), during their first breeding season (~1‐year‐old), and during their further adult life (≥2 years old). Because of their slow pace of life, tropical stonechats are expected to prioritize somatic maintenance over fecundity. Thus, we expected longer telomeres in tropical compared to temperate stonechats. Further, because of their higher juvenile survival and extended parental care we expected a shallower decrease in telomere length during the first year of life in tropical compared to temperate stonechats.

## 2 METHODS

Blood samples were collected from two phylogenetically closely related stonechat species (Table 1) during their respective breeding seasons (nestlings [day 8–14 post‐hatch], first year breeding [~1‐year‐old] and adult males [≥2 years old]) or just after the breeding season (first year pre‐breeding males (3–6 months post‐hatch). In addition, in tropical stonechats, we were occasionally able to catch fledglings (>16 days old), which had only recently left their nest and were still in their juvenile plumage and cared for by their parents. Stonechats were sampled in tropical East Africa (*Saxicola torquatus axillaris,* four populations, latitudes 0°–4°S, altitudinal range: 1,376–2,500 m at sea level [asl], sampled from 2012 to 2014) and Europe (*S. rubicola*, three populations, latitudes 37°–51°N, altitudinal range: 15–40 m asl, sampled from 2013 to 2015, Table 1). While East African stonechats are residents, European populations vary in migratory strategy from short‐distance migrants to residents (Table 1). Samples from some of these birds were also used to study baseline and stress‐induced corticosterone concentrations of tropical and temperate stonechats during the breeding season (Apfelbeck, Helm, et al., 2017). All samples were analyzed with telomere restriction fragment analysis (TRF) and we restricted our analysis to males to optimize sample sizes across species and age classes.

**Table 1:** Sample sizes of male stonechats by regions and age categories

Region	Population, latitude, longitude, altitude, migratory strategy	Nestling	Fledgling (before post‐juvenile moult)	First year, pre‐breeding (after post‐juvenile moult)	First year, breeding (before first post‐nuptial moult)	≥2nd year, breeding
East Africa, *Saxicola torquatus axillaris*	Kinangop, 0°37′N, 36°29′E, 2,470 asl, resident	15 (from 12 nests)			5	19
	MountMeru, 3°16′S, 36°51′E, 1,573 m asl, resident		1	3 (4–6 months old)		
	Taita Hills, 3°22′S, 38°20′E, 1,400 m asl, resident		2	4 (4–6 months old)		
	Monduli, 3°14′S, 36°25′E, 1,923 m asl, resident		2		7	
Europe, *Saxicola rubicola*	Ireland, 51°49′N, 8°00′W, 21 m asl, partial migratory	10 (from 5 nests)			13	1
	Spain, 37°39′N, 5°34′W, 40 m asl, resident				14	7
	Germany, 51°47′N, 6°01′E, 15 m asl, migratory			12 (3–6 months old)		6

Note

For more detailed information on the sites (see Apfelbeck, Helm, et al., 2017).

### 2.1 Capture methods

Male stonechats were caught between 07:00 hr and 18:00 hr with baited clap net traps but also in some cases additionally lured by a mounted decoy and playback. Traps were observed continuously and upon capture birds were immediately removed from the traps. Nestlings were bled during the last third of the nestling stage (i.e., between day 8 and 14 post‐hatch). Pre‐breeding first‐year males were caught 3–6 months (see Table 1) after their likely hatching dates, after completing post‐juvenile moult, but before their first breeding season. Breeding first‐year males were caught during their first breeding season before their first post‐nuptial moult. All males were measured (weight, tarsus, and wing length), checked for moult, ringed with a numbered aluminum ring and a combination of three color rings and were then released back into their territories. We determined the age of all individuals caught as either in their first year or as adults (≥2 years) based on feather moult pattern of the wings (Flinks, 1994). Nestlings and fledglings were ringed with a numbered aluminum ring only.

### 2.2 Blood sampling

Blood samples (~120 µl) were taken within 3 min of capture by venipuncture of the wing vein or (less often) with an insulin syringe from the jugular vein and collected into heparinized capillaries. Plasma was immediately separated by centrifugation with a Compur Minicentrifuge (Bayer Diagnostics) or a Spectrafuge Mini Laboratory Centrifuge (Labnet International, Inc.) and plasma and blood cells were stored separately in pure ethanol or in few cases in Queens buffer. In the case of fledglings and nestlings, whole blood (~40 µl) was directly stored in pure ethanol. Correct measurement of telomere length across samples relies on DNA integrity and recent studies have shown that DNA integrity can depend on the way samples were stored (Nussey et al., 2014; Reichert et al., 2017), and thus, preferentially samples should be treated similarly across groups. Although in our study storage method varied across samples, we are confident that this did not influence our results as DNA integrity was checked for each sample by standard gel electrophoresis (Kimura et al., 2010). Samples that showed signs of degradation were not included in the TRF assay. Upon return from the field, samples were stored at ~2°C whenever possible. During periods of transportation, samples were stored at room temperature.

### 2.3 Sex determination of nestling and fledgling samples

Molecular sexing was carried out by amplification of the chromodomain‐helicase‐DNA binding (CHD) genes in 10 µl PCR reactions following the standard procedure described in Fridolfsson and Ellegren (1999) and Griffiths, Double, Orr, and Dawson (1998).

### 2.4 Telomere length assay

Telomeres were measured with the TRF assay, and the procedure was carried out according to previous studies (Haussmann & Mauck, 2008; Marchetto et al., 2016). Briefly, DNA was extracted from packed blood cells using the Puregene Blood Core Kit B following the manufacturer's specifications (Qiagen). DNA integrity was assessed through the use of integrity gels (Nussey et al., 2014), and telomeres of high integrity DNA samples were then measured using the TRF assay. A 10 µg quantity of DNA was digested using 1.0 ml of RsaI (New England Biolabs, R0167L) and 0.2 ml of HinfI (New England Biolabs, R0155M) in CutSmart Buffer (New England Biolabs, B7204S) overnight at 37°C. The digested DNA was separated using pulsed‐field gel electrophoresis (3 V/cm, 0.5‐ to 7.0‐s switch times, 14°C) for 19 hr on a 0.8% nondenaturing agarose gel. The gel was then dried without heating and hybridized overnight with a ^32^P‐labeled oligo (5′CCCTAA‐3′) that binds to the 3′ overhang of telomeres. Hybridized gels were placed on a phosphor screen (Amersham Biosciences, Buckinghamshire, UK), which was scanned on a Storm 540 Variable Mode Imager (Amersham Biosciences). We used densitometry (ImageQuant 5.03v and ImageJ 1.42q) to determine the position and strength of the radioactive signal in each of the lanes compared to the molecular marker (1 kb DNA Extension Ladder; Invitrogen, CA). The background was fixed as the nadir of the low‐MW region on the gel (<1 kb). Samples were distributed among six gels and mixed by population and age class. One stonechat sample was run three times on each gel to determine intra‐ and inter‐gel coefficients of variation, which were 4.86% and 7.54%, respectively.

### 2.5 Statistical analysis

Data were analyzed within the R environment (R version 3.2.2; R Core Team, 2016) and the packages arm (Gelman & Su, 2018), JAGS (Plummer, 2003), and runjags (Denwood, 2016). Linear models were used to determine whether variation in mean telomere length was related to breeding region (tropical, temperate), age class or the interaction between breeding region and age class. We tested whether tropical stonechats had longer telomeres than temperate stonechats and whether differences between taxa declined with age by comparing tropical and temperate male stonechats in different age classes (nestling, first year pre‐breeding, first year breeding, ≥2 years). We applied linear models using tropical nestlings as reference level. Body mass was included as a covariate in the initial model, but dropped in the final model as it did not detectably explain variance in the data. Because samples from tropical stonechats covered the widest range of age classes, we ran a separate linear model on tropical stonechats including the additional factor level “fledglings.”

We chose a Bayesian approach to draw inferences from the models. Bayesian statistics estimate probability distributions of the parameters in the model (i.e., posterior distributions) given the data and prior knowledge about the distribution of the data (specified as priors) (Korner‐Nievergelt, Roth, Felten, & Guélat, 2015). Model parameters were estimated as the mean of their posterior distributions, and the 2.5% and 97.5% upper and lower margins of the credible intervals. Minimally informative priors for both mean (dnorm [0, 10^−^^6^]) and variance (dgamma [0.001, 0.001]) parameters were used, that is, we assumed no prior knowledge about the factors in our models. Marcov Chain Monte Carlo simulations were checked for convergence of chains using trace plots and psrf values (Brooks & Gelman, 1997). Effective sample sizes were >15,000 in all cases. Model residuals were graphically checked for violations of model assumptions (normality, heteroscedasticity, autocorrelations) (Korner‐Nievergelt et al., 2015). Data are presented as means and their 95% Bayesian credible intervals in figures and as the difference and 95% Bayesian credible interval (in squared brackets) from the mean intercept in tables. Bayesian statistics do not produce test statistics or *p*‐values; however, when the Bayesian 95% credible interval of the difference between two means does not include zero, this can be interpreted as a detectable difference (Held & Sabanés Bové, 2014).

## 3 RESULTS

**Figure 2:**
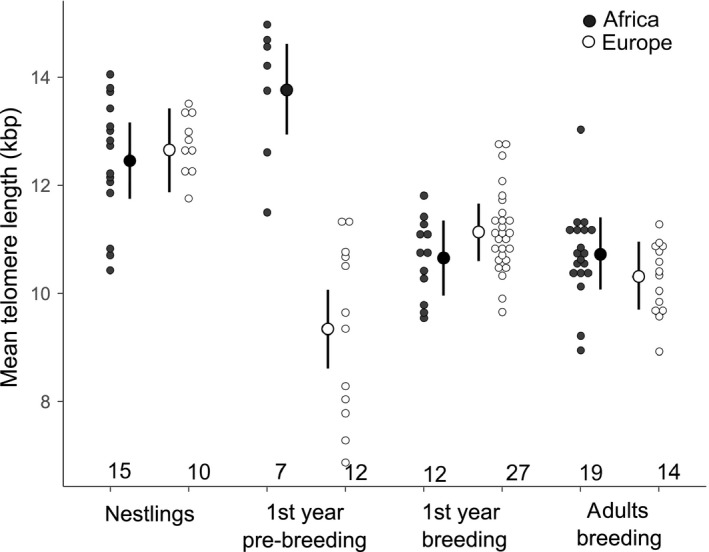
Mean telomere length (kbp) of tropical and temperate male stonechats (*Saxicola *ssp.*) *in different age classes. Depicted are posterior means and their 95% Bayesian credible intervals (errors bars). Smaller dots represent data points from individuals. Sample sizes are given below dot plots

Overall, temperate and tropical male stonechats showed similar mean telomere lengths (Table 2, Figure 2). In particular, temperate and tropical males had similar telomere lengths as nestlings, first year and adult breeders. Breeding males (first year and adults) had shorter telomeres than nestlings both in tropical and in temperate stonechats (negative differences from intercept for first year and adult tropical breeders, no additional detectable difference for first year and adult temperate breeders, Table 2, Figure 2). However, telomere lengths of tropical and temperate stonechats differed in their first year of life. While tropical first year pre‐breeding males had longer telomeres than nestlings (positive difference from intercept), temperate first year pre‐breeding males had shorter telomeres than nestlings (negative difference from intercept, Table 2, Figure 2). Analysis of tropical stonechats across the whole range of age classes revealed that average telomere length was similar in nestlings and fledglings of tropical stonechats (Table 3). Similar telomere lengths of tropical and temperate first year breeding males indicate a decrease in mean telomere length in tropical stonechats, but an increase in mean telomere length in temperate stonechats from first year pre‐breeding to first year breeding males. To confirm this increase in temperate stonechats from first year pre‐breeding to first year breeding males, we ran a linear model for temperate stonechats restricted to the age classes first year pre‐breeding, first year breeding and adult. This post hoc analysis confirmed a detectable increase from first year pre‐breeding to first year breeding in temperate males (intercept first year pre‐breeding: 9.3 [8.7, 10.0], difference first year breeding 1.3 [0.5, 2.0], difference adult 0.9 [−0.04, 1.8]).

**Table 2:** Mean telomere length of male stonechats (*Saxicola *ssp.) in relation to breeding region (tropical, temperate) and age class (nestling, first year pre‐breeding, first year breeding, adult breeding)

Factor level	Estimates (differences from the intercept) and 95% credible intervals, (mean telomere length, kpb)	Estimates and 95% credible intervals (mean telomere length, kbp)
Intercept: tropical, nestling	12.5 [11.9, 13.0]	12.5 [11.9, 13.0]
Tropical, first year pre‐breeding	**1.3 [0.4, 2.2]**	**13.8 [12.3, 15.2]**
Tropical, first year breeding	**−1.8 [−2.6, −1.0]**	**10.7 [9.3, 12.0]**
Tropical, adult breeding	**−1.7 [−2.4, −1.1]**	**10.8 [9.5, 11.9]**
Temperate, nestling	0.3 [−0.5, 1.1]	12.8 [11.4, 14.1]
Temperate, first year pre‐breeding	**−4.7 [−6.0, −3.5]**	**9.0 [6.5, 11.6]**
Temperate, first year breeding	0.2 [−0.8, 1.2]	10.9 [8.6, 13.5]
Temperate, adult breeding	−0.8 [−1.8, 0.3]	10.0 [7.8, 12.1]

Note

The second column shows the estimated difference from the intercept. In this case the reference level was “tropical nestlings.” The third column shows the mean estimates for each factor level, which were calculated from column 2. As the reference level was “tropical nestlings,” for temperate birds the estimated difference has to be added to the estimate obtained for tropical birds for each age class. When 0 (zero) is not included in the credible intervals there is an effect of this parameter on the dependent variable (shown in bold).

**Table 3:** Mean telomere length of tropical stonechats in relation to age class (nestling, fledgling, first year pre‐breeding, first year breeding, adult)

Factor level	Estimates (differences from the intercept) and 95% credible intervals (mean telomere length [kbp])
Intercept: nestling	12.5 [11.9, 13.0]
Fledgling	0.6 [−0.5, 1.6]
First year nonbreeding	**1.3 [0.4, 2.2]**
First year breeding	**−1.8 [−2.6, −1.0]**
Adult breeding	**−1.7 [−2.4, −1.0]**

Note

Estimates are relative to the intercept as reference level, in this case nestlings. When 0 (zero) is not included in the credible intervals there is an effect of this parameter on the dependent variable (shown in bold).

## 4 DISCUSSION

Our data provide first evidence that distinct selective pressures in tropical and temperate environments may be reflected in diverging patterns of telomere loss between age classes. Similar to other studies (e.g., Spurgin et al., 2018), we find that mean telomere length decreased fastest during the first year of life in temperate stonechats. In contrast, in tropical stonechats mean telomere length increased initially from nestlings to fledglings to first year pre‐breeding before dropping in first year breeding males. This suggests that tropical compared to temperate stonechats may either experience lower levels of telomere loss and/or more pronounced differential survival of individuals with long telomeres during their first year of life.

In tropical birds, the post‐fledging period has emerged as a critical period during reproduction that may have a considerable impact on the fitness of tropical birds. Tropical birds experience high levels of nest predation (Martin, 1992), but high levels of adult survival. Therefore, tropical parents may raise small clutches in favor of extended offspring care (Russell, Yom‐Tov, & Geffen, 2004; Tarwater, Ricklefs, Maddox, & Brawn, 2011), which has been shown to increase fledgling survival (Grüebler & Naef‐Daenzer, 2010). Tropical stonechats lay on average smaller clutches (3) than temperate stonechats (5) and care for fledglings for several weeks after fledging, often allowing juveniles to remain on their territories (Dittami & Gwinner, 1985; Scheuerlein, Van't Hof, & Gwinner, 2001). In addition, especially under high predation pressure, stonechats often skip a second clutch in favor of their fledglings (Scheuerlein et al., 2001). Also, as has been shown for other species, parents may actively favor the strongest of their fledglings (Barrios‐Miller & Siefferman, 2013). Thus, while survival probabilities post‐fledging are in general low (Naef‐Daenzer & Grüebler, 2016), high‐quality fledglings may have a higher survival probability in tropical than in temperate stonechats, leading to longer telomeres in tropical compared to temperate juveniles.

In addition, as environmental conditions and parental care during growth can influence telomere loss and maintenance (e.g., Costanzo et al., 2017), extended parental care may create more favorable conditions for tropical fledglings and juveniles that may allow them to maintain their telomeres better than temperate ones. A number of recent studies in temperate songbirds during the nestling period have shown that the rearing environment has an influence on telomere attrition rates in early life (e.g., Salmon et al., 2016; Soler et al., 2017). For example, growing up in large broods, high begging effort and low food availability hasten telomere loss in nestlings (Costanzo et al., 2017; Nettle et al., 2017; Young et al., 2017). Furthermore, studies in jackdaws, *Corvus monedula*, and great tits, *Parus major*, demonstrated that the rate of telomere loss early in life matters, as individuals that recruited into the breeding population in the following year showed lower rates of telomere loss and longer telomeres as nestlings than those that did not survive their first year (Boonekamp et al., 2014; Salmon et al., 2017). The effect of extended parental care in tropical birds on telomere dynamics in fledglings should be further investigated, for example through brood size manipulation experiments.

In contrast to telomere length in juveniles, telomere length at the end of the nestling stage did not differ between temperate and tropical stonechats despite smaller clutches in tropical stonechats, which favor increased feeding rates per offspring (Martin, 2015). As ground‐nesting birds in open habitat, high nest predation rates may favor fast growth and consequently similar telomere loss during development in tropical and temperate environments. In tropical stonechats, frequent presence of predators reduced the growth rate of nestlings (Scheuerlein & Gwinner, 2006), highlighting that lower growth rates in stonechats may not represent a mechanism for (supposedly) slower aging of tropical stonechats. However, direct comparisons of growth rates in nestlings of tropical and temperate stonechats are necessary to further clarify this issue.

Selective disappearance of individuals with short telomeres during the first year of life also seems to take place in temperate stonechats, albeit later than in tropical stonechats, potentially during the first migration and overwinter period. Migration or the winter period are especially challenging for inexperienced, subordinate juvenile birds and accordingly mortality during migration and winter is higher for juveniles than for adults (Ekman, 1984; Rotics et al., 2016). Only the highest quality individuals with the longest telomeres may be able to survive, which may lead to longer telomeres in first year breeding males compared to males caught before their first winter (first year pre‐breeding). Thus, mean telomere length across age classes may indicate when selective disappearance of low‐quality individuals with short telomeres is most likely to occur.

In contrast to our predictions, tropical and temperate stonechats had telomeres of similar length during their first breeding season and as adults. Reproduction, especially parental care, is energetically costly (Nilsson, 2002), can lead to oxidative DNA damage (Noguera, 2017) and potentially increased telomere loss (Heidinger et al., 2012; Reichert et al., 2014). Our previous comparative studies on hormone levels and mating behavior in tropical and temperate stonechats have shown that territorial aggression is accompanied by a peak in testosterone and corticosterone concentrations during nest‐building in both temperate and tropical male stonechats (Apfelbeck, Helm, et al., 2017; Apfelbeck, Mortega, et al., 2017). Thus, tropical and temperate male stonechats engage similarly in costly mating behaviors which may potentially affect telomere length. Furthermore, in adult birds, the exact age of individuals could not be determined and we probably sampled across their life expectancy, which might mask selective disappearance or lower telomere attrition in tropical birds and account for similar telomere lengths in tropical and temperate adult stonechats. To determine whether lower mortality in benign tropical environments favors selective disappearance of individuals with short telomeres, studies in populations, in which the age of adult individuals is known, are needed.

In this study, samples from different age classes were collected from different individuals. Longitudinal studies show that telomere shortening rates are often higher within individuals than telomere shortening rates at the population level (Salomons et al., 2009). Thus, in studies based on cross‐sectional samples, it is difficult to disentangle the effects of telomere attrition and selective disappearance for different age classes. Also, we were not able to sample all age classes in all populations. Thus, differences between populations and taxa in breeding altitude and migratory strategy may have confounded our results as they may affect telomere dynamics (Bauer, Heidinger, Ketterson, & Greives, 2016; Stier et al., 2016). For stonechats, the effects of breeding latitude, altitude, and migratory strategy are not easily separable as tropical stonechats breed at high altitudes and are residents, while breeding altitudes and migratory strategies of European stonechats vary more. However, a resident lifestyle is commonly found in the tropics and is actually part of a slow life history (Dobson, 2012). Variation between European stonechat populations can be used to disentangle potential effects of breeding altitude and migratory strategy on telomere dynamics in future studies.

Nevertheless, our data indicate that different life history strategies of tropical and temperate birds may be reflected in distinct patterns of telomere loss during the first year of life and can be the basis for future in‐depth studies on variation in telomere dynamics between tropical and temperate environments. To separate the relative importance of telomere attrition and selective disappearance during the first year of life, future studies should measure telomere length and survival of tropical and temperate nestlings and fledglings longitudinally in different stonechat populations within tropical and temperate breeding regions and with different migratory strategies. Ideally, the experiments should be extended to other tropical and temperate species to determine whether the patterns found here are indeed a consequence of life history variation between tropical and temperate environments.

## 5 CONCLUSIONS

To the best of our knowledge, this is one of the first studies to compare mean telomere length across several age classes in closely related species that breed in tropical and temperate environments and differ in their pace of life. As indicated by previous interspecific studies, our results suggest that variation in life history and life span may be reflected in different patterns of telomere loss between species rather than absolute telomere length. Our data reveal that mean telomere length across age classes may indicate during which life‐cycle phases individuals with short telomeres, and thus of potentially low quality, are most likely to disappear from a population. These patterns closely fit with expectations from life history theory and match variation in parental behavior and juvenile mortality between tropical and temperate birds.

## ETHICAL APPROVAL

All experimental procedures were approved by the governmental authorities of the respective countries (Germany: Regional Government of North Rhine Westphalia and Vogelwarte Helgoland License No. 0147; Ireland: License No. C092/2013; Kenya: National Commission for Science, Technology and Innovation (NACOSTI); Spain: Regional Governments of Junta de Castilla y León (EP‐CYL‐33–2013), Andalucía (35–36/13) and the Canary Islands (2012/0710); Tanzania: Tanzania Wildlife Research Institute (TAWIRI) and Tanzania Commission for Science and Technology (COSTECH)).

## CONFLICT OF INTEREST

None declared.

## AUTHOR'S CONTRIBUTION

BA and BH conceived of the study. BA, HF, JCI, KGM, PS collected the field data. BA and ZS conducted the molecular laboratory work. WB, KG, and MH supported and supervised the laboratory work. BA carried out the statistical analysis and drafted the manuscript. MH and BH helped drafting the manuscript. All authors gave final approval for publication.

## DATA ACCESSIBILITY

The dataset supporting this article has been uploaded as Supporting Information.
